# The Landscape of Immunotherapy Resistance in NSCLC

**DOI:** 10.3389/fonc.2022.817548

**Published:** 2022-04-20

**Authors:** Daniele Frisone, Alex Friedlaender, Alfredo Addeo, Petros Tsantoulis

**Affiliations:** ^1^ Department of Oncology, Geneva University Hospital, Geneva, Switzerland; ^2^ Department of Oncology, Clinique Generale Beaulieu, Geneva, Switzerland; ^3^ Department of Oncology, Faculty of Medicine, Geneva University, Geneva, Switzerland

**Keywords:** immunotherapy, resistance, checkpoint inhibitor, NSCLC, lung cancer

## Abstract

Lung cancer is the leading cause of cancer mortality worldwide. Immunotherapy has demonstrated clinically significant benefit for non-small-cell lung cancer, but innate (primary) or acquired resistance remains a challenge. Criteria for a uniform clinical definition of acquired resistance have been recently proposed in order to harmonize the design of future clinical trials. Several mechanisms of resistance are now well-described, including the lack of tumor antigens, defective antigen presentation, modulation of critical cellular pathways, epigenetic changes, and changes in the tumor microenvironment. Host-related factors, such as the microbiome and the state of immunity, have also been examined. New compounds and treatment strategies are being developed to target these mechanisms with the goal of maximizing the benefit derived from immunotherapy. Here we review the definitions of resistance to immunotherapy, examine its underlying mechanisms and potential corresponding treatment strategies. We focus on recently published clinical trials and trials that are expected to deliver results soon. Finally, we gather insights from recent preclinical discoveries that may translate to clinical application in the future.

## Introduction

Lung cancer is the leading cause of cancer mortality, with 1.750.000 estimated deaths in 2018 worldwide (1). By far, the most common type of lung cancer is non-small-cell lung cancer (NSCLC) (2). The advent of immune checkpoint inhibitors (ICIs) completely changed the therapeutic landscape of NSCLC, but the majority of lung cancer patients eventually progress during immunotherapy (3–5). Chemo-immunotherapy combinations are associated with improved outcomes, especially in patients with PD-L1 < 50% NSCLC (6–8). Even with chemo-immunotherapy combinations, a significant proportion of patients does not respond to treatment or responds only for a limited period, with mPFS varying from 4 to 10 months in different trials (9).

Resistance to immunotherapy is not fully understood. We briefly review recently acquired knowledge in this domain. We then examine the development of treatment strategies and compounds that aim to overcome resistance based on this knowledge, including results from early clinical trials and preliminary findings. Finally, we go through pivotal trials that are currently underway and which will hopefully provide additional insight in the next few years.

## Main Mechanisms of Resistance and Definitions

A recent review by Wang et al. classified resistance to immunotherapy based on the timing of its development, the characteristics of the cancer cell, and the type of immune infiltrate (9). With respect to timing, primary resistance is defined as disease progression during first-line ICIs, and acquired resistance is defined as tumor progression after initial disease control (10). A recent revised definition of acquired resistance to immunotherapy in NSCLC excludes stable disease (SD) since SD is too heterogeneous. For example, patients who have a slowly growing disease, unresponsive to immunotherapy, are considered to have SD at the first imaging evaluation but should be considered as primarily resistant to immunotherapy (11). When the cancer cell itself is considered, we can identify intrinsic resistance, which is related to genomic or proteomic features, and extrinsic resistance, which is modulated by the tumor microenvironment, including the immune cells. Finally, the spatial distribution of immune cells can create distinct patterns with complete absence of immune cells (immune desert), abundant intratumoral immune cells within the tumor and at the periphery (inflamed pattern), or absence of immune cells in the tumor bed, with immune cells only at the invasive margin (immune excluded tumors) (12).

**Figure 1:**
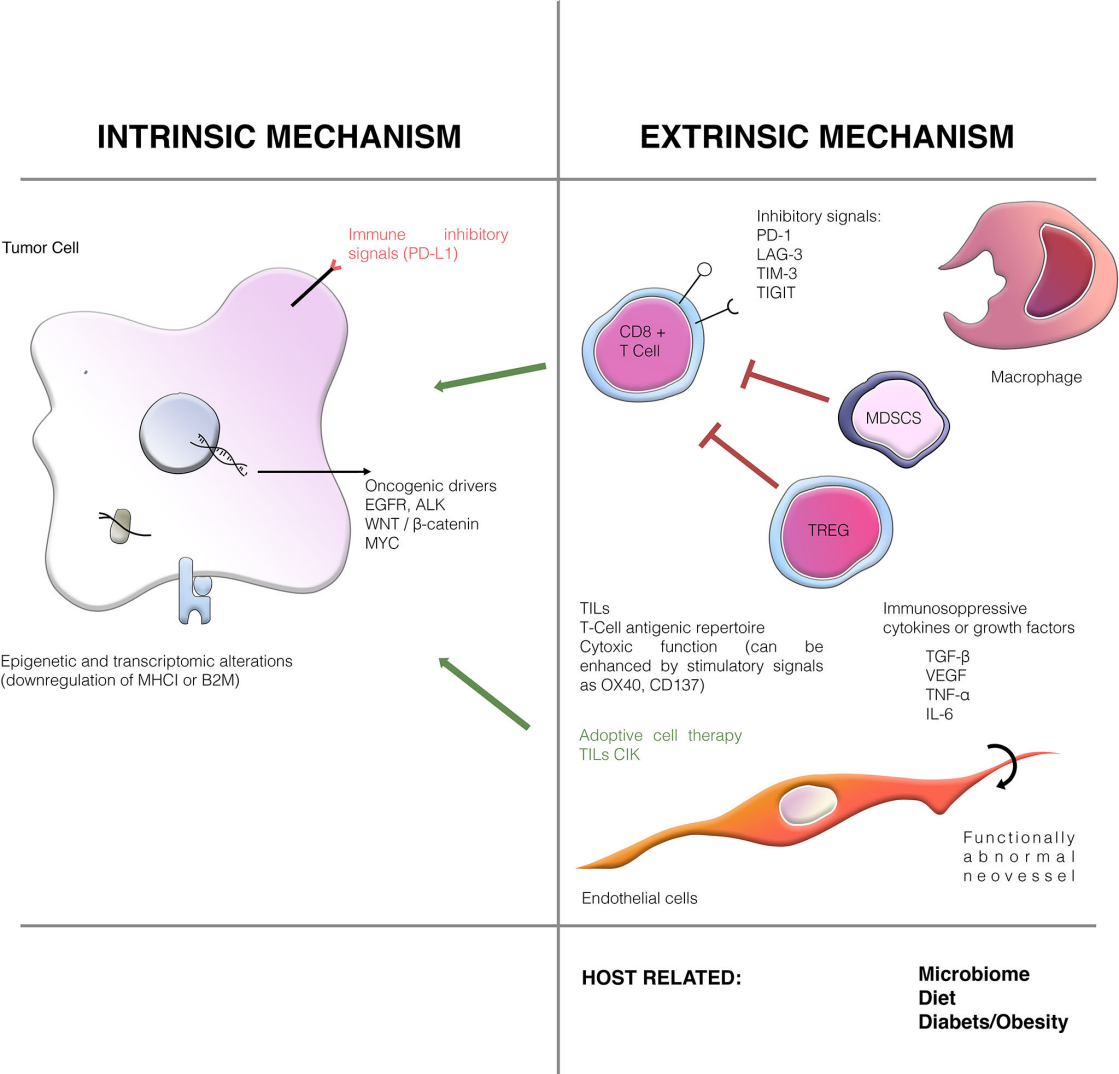
Mechanisms of resistance to immunotherapy and main molecular pathways involved.

The distinction between primary and secondary resistance is applicable in clinical trials and for clinical purposes but cannot be used to guide treatment selection and does not offer any mechanistic insight for the development of more effective therapies or biomarkers. In the following sections, we will focus on resistance mechanisms related to the cancer cell and its microenvironment (**Figure 1**). We do not review host-related mechanisms such as microbiome or diet, that could influence the effect of immune-based therapies.

### Intrinsic Mechanisms

Intrinsic features of the cancer cell, such as the genomic and proteomic profile, are the main drivers of resistance to treatment. Immune checkpoints are molecules that modulate inhibitory immune-signaling pathways, regulating the duration of immune responses as well as self-tolerance (13). These pathways are essential in physiological immune responses and avoid uncontrolled reactions and collateral damage. The expression of inhibitory signals (checkpoints) by the cancer cell is an essential mechanism of immune evasion and can involve a number of inhibitory molecules such as PD-L1 (CD274), IDO, LAG3, TIM-3, VISTA, and others (14). Oncogene addiction has also been associated with primary resistance to immunotherapy (9). Not all oncogenic drivers induce resistance to immunotherapy. For instance, while *EGFR* mutations or *ALK* fusions predict resistance to ICIs regardless of PD-L1 expression, BRAF and, especially, KRAS mutations do not (15–17). This may stem from a different tumor microenvironment and higher neoantigen load from tobacco smoke exposure in patients with BRAF/KRAS mutations (18). Preliminary trials combining ICIs and EGFR inhibitors showed increased liver and lung toxicity. Given the low efficacy of ICIs in *EGFR* mutant NSCLC, further trials in this space have been stopped (19).

Other intracellular signaling pathways and genes can also contribute to immunotherapy resistance, either directly or by influencing the tumor microenvironment. Genes commonly implicated in NSCLC include *STK11/LKB1, KEAP1, MYC, TP53, PTEN*, and the Wnt/β-catenin pathway (20). STK11 mutations in NSCLC may be associated with worse outcomes with ICIs since the inactivation of this serine threonine kinase involved in various cellular functions is associated with a low T lymphocytes tumor infiltration and a “cold” tumor microenvironment (21, 22). However, it is not clear whether the impact of STK11 mutations is predictive of poor response to ICIs specifically, or simply prognostic of aggressive tumor evolution (23–25). Furthermore, in an exploratory analysis of the KEYNOTE-042 trial comparing single-agent pembrolizumab to chemotherapy, STK11 mutations did not appear to impact the efficacy of ICIs, while they were associated with a worse prognosis in the chemotherapy cohort (26). *KEAP1* mutations are also quite common and have been associated with poor prognosis, but there is conflicting evidence regarding their interaction with immunotherapy (27, 28). Common co-occurring mutations involving *KEAP1, PBRM1, SMARCA4*, and *STK11* may synergistically explain immunogenomic differences acting against immune response (29). Mutations in *TP53* also are more common in patients responding to immunotherapy although the exact mechanism is not clear (30). Recently, loss of 9p21 was shown to confer a cold immune phenotype with reduced lymphocyte infiltrates and worse outcomes with immunotherapy (31). Unfortunately, there is no satisfactory treatment directed at these alterations, and no clinical trial results are available with targeted treatment of these alterations alone or in combination with ICIs (32).

Epigenetic and transcriptomic alterations may play a role in conferring intrinsic resistance to immunotherapy to tumor cells, with inflammatory and mesenchymal phenotypes being recently associated with primary resistance in melanoma (9). For instance, the downregulation of MHC I or of some of its components, such as B2M, is a frequent mechanism of escape to T cell killing that can be due to transcriptomic or epigenetic alterations (33, 34).

### Extrinsic Mechanisms

Competent cytotoxic T-cells are crucial for antitumoral immune response. First, the antigenic repertoire of the T-cell receptors (TCRs) must react with the immune profile of the tumor, including expressed neoantigens (9, 35).. Second, the T-cells must infiltrate the tumor bed and physically interact with cancer cells. The presence of infiltrating CD8+ T-cells is positively correlated with more favorable outcomes in NSCLC (36). Third, T-cells must maintain their proliferative capacity and cytotoxic potential and avoid a dysfunctional “exhausted” state, which can be driven by the activity of regulatory T (Treg) cells, chemokines, and growth factors (33, 37).

An immunosuppressive TME is an important cause of immune resistance. The production of immunosuppressive cytokines or growth factors, such as VEGF and TGF-β (9), by the TME is one of the best-studied resistance mechanisms. Hypoxia, a common feature of tumors, can induce the expression of VEGF through HIF-1α. VEGF acts on endothelial cells to promote tumor neo-angiogenesis, but also has immunosuppressive properties, leading to a decrease of cytotoxic T-cells in favor of immunosuppressive Tregs and other myeloid-derived suppressor cells (MDSCs) (9, 20). In fact, tumor-induced neoangiogenesis is associated with structurally and functionally abnormal neovessels, with endothelial cells, pericytes and immune cells being involved in a complex crosstalk (38). Due to the abnormal vessel structure, the process of rolling, adhesion and transmigration becomes more difficult for immune cells and fewer of them are able to leave the bloodstream and infiltrate the tumor. Moreover, functionally abnormal vessels also induce hypoxia and acidosis which can have an immunosuppressive influence on T cells and on macophages. It has been claimed that the use of anti-VEGFR molecule with the aim of “vasculature normalization” more than antiangiogenesis, can result in polarizing tumor-infiltrating immune cells toward stimulatory phenotypes (39).

In this complex TME landscape, TGF-β also plays a vital role in promoting cancer cell invasion and metastases, also contributing to an immunosuppressive milieu by suppressing dendritic cell differentiation and migration, preventing Th1 and Th2 differentiation while promoting Treg programs (40–42).

## Overcoming Immunotherapy Resistance

### Targeting Co-Inhibitory Signals

As discussed above, multiple signals inhibiting T-cells can be expressed by the cancer cell or the environment. Targeting co-inhibitory signals beyond PD-1/PD-L1 may help overcome resistance, but with the exception of combined treatment directed against PD-1/PD-L1 and CTLA4, the experience in NSCLC is limited.

The Checkmate 227 study showed a significant benefit of ipilimumab combined with nivolumab over platinum-based chemotherapy in terms of overall survival, but adding ipilimumab to pembrolizumab in first-line setting NSCLC with PD-1≥50% did not improve survival over pembrolizumab alone (43, 44). A prospective study with tremelimumab, another antibody directed against CTLA4, and durvalumab in patients progressing on anti-PD-1 immunotherapy has been recently published, showing a lack of clinical activity in this setting (ORR: 0% for acquired resistance cohort, with a mPFS of 2.1 months) (45).

Five co-inhibitory signals other than CTLA4 have been targeted in clinical trials: LAG-3, TIM-3, TIGIT, VISTA, and Siglec-15 (**Table 1**).

**Table 1:** Results of clinical trial for new agents targeting co-inhibitory signals on T cells and epigenetic alterations.

Trial name/code	Setting	Phase	Molecule	N° of NSCLC patients	ORR (CR)	DCR	Median PFS	Median OS
ENCORE 601	Pretreated with ICIs	I	Entinostat (histone deacetylase inhibitor)+ pembrolizumab	71	9,2%	–	2,8 mo	11,2 mo
NCT02608268	Pretreated with ICIs	II	MBG-453 (anti-TIM3) + spartalizumab	17	0/17	7/17 (41,2%)	–	–
CITYSCAPE (NCT03563716)	First line PD-L1+	II random	Tiragolumab (anti-TIGIT) + atezo vs Placebo + atezolizumab	135	37,3% vs 20,6%	–	5,6 mo vs 3,9 mo	–
CTRI/2017/12/011026	Pretreated, ICIs naïve	II	CA-170 (dual VISTA and PD-L1 oral inhibitor) 400 mg	8 non squamous	0/8	6/8 (75%)	19,5 weeks	–
NCT03665285	Pretreated with ICIs	I	NC318 (anti Siglec-15)	13	2/10 (1)	6/10 (60%)	–	–
NCT03667716	Pretreated with ICIs	I	COM701 (PVRIG inhibitor) +/- Nivolumab	?	0	6/20 (3 NSCLC)	–	–

LAG-3 (Lymphocyte-Activation Gene-3) is a transmembrane protein that binds to the MHC class II complex. LAG-3 overexpression has been associated with T-lymphocyte exhaustion and resistance to anti-PD-1/PD-L1 antibodies (46, 47). In first-line advanced melanoma, a positive phase III trial has been published with relatlimab in combination with nivolumab, with HR of 0,75 for PFS in favor of the combination arm versus single-agent nivolumab and no clinically significant increase in toxicity (48). Results from phase I and phase II trials with multiple histologies have been presented as abstracts, but there’s no specific data on NSCLC at the moment (49, 50).

TIM-3 (T-cell immunoglobulin and mucin domain 3) is a type I glycoprotein with an extracellular Ig V domain. It has immune-modulatory properties, and its overexpression is linked to a worse prognosis (51, 52). Upregulation of this molecule has also been linked to acquired resistance to anti-PD-1 antibodies so that combination therapy has been designed (51, 53). Few results of clinical trials with TIM-3 inhibitors alone or in combination with PD-1 have been presented. An abstract presentation of a phase II trial showed limited clinical benefit in the setting of progression after previous ICIs in NSCLC or melanoma patients (54).

TIGIT (T cell immunoglobulin and ITIM domain) is another co-inhibitory transmembrane receptor crucial for immune tolerance, expressed by T cells and NK (47). Tiragolumab, a TIGIT inhibitor, is the only new generation ICI for which randomized data have been published, although in a phase II trial (20, 47). The CITYSCAPE trial randomized 135 treatment naïve patients, with advanced PD-L1 positive NSCLC, to atezolizumab vs. tiragolumab+atezolizumab, obtaining good ORR and PFS, especially in the PD-L1 ≥50% (66% for combination versus 24% for atezolizumab) (55). The toxicity profile was good with a rate of G3 or higher events similar in the two arms. A phase III trial (NCT04294810) is ongoing for patients with PD-L1 TPS≥50%. Finally, a molecule targeting the co-inhibitory receptor PVRIG, a member of the same family as TIGIT, has demonstrated good tolerability and some encouraging activity in pretreated patients, but just three disease stabilizations in the NSCLC patient cohort (56, 57).

VISTA (V-domain immunoglobulin suppressor of T –cell activation) is a type I transmembrane protein able to suppress T-cell activation, with sequence homology with PD-1 and PD-L1 (58). This has permitted the development of an oral dual inhibitor (PD-L1 and VISTA) CA-170, which showed an interesting activity and safety profile in pretreated but ICI naive NSCLC, with a dosage of 400 mg daily (59, 60).

Siglec-15 is a protein expressed mainly by myeloid cells and known to have a role in bone metabolism. Siglec-15 has been recently described as a possible immune-escape mechanism (61). The first-in-human study for an anti-Siglec-15 antibody (NC318) reported some signal of efficacy with responses in 2/13 ICI-pretreated NSCLC patients (20).

### Enhancing Co-Stimulatory Signals

Enhancing the immune response of T and NK cells is another promising way of overcoming immunotherapy resistance and is supported by robust preliminary clinical data. For NSCLC patients, OX-40, CD137, and the use of cytokine superagonists such as IL-15 are currently investigated (**Table 2**). OX-40 (or CD134) and CD137 (or 4-1BB) are surface glycoproteins expressed by some immune cells, including T-cells and APCs. They are induced by antigenic stimulation and are considered markers of effector T-cells. They are co-stimulatory molecules that can mediate immune-cell activation, making them attractive therapeutic targets in cancer (62).

**Table 2:** Results of clinical trials for new agents targeting co-stimulatory molecules on T cells and cellular therapy.

Trial name/code	Setting	Phase	Molecule	N° of NSCLC patients	ORR (CR)	DCR	Median PFS	Median OS
NCT02315066	Pretreated with ICIs	I	PF-8600 (OX40 agonist) + utomilumab (CD137 agonist)	20	5%	40%	–	–
QUILT 3.055 (NCT03228667)	Pretreated with ICIs	II	N803 (IL-15 superagonist + ICI)	81	8%	59%	3,9 mo	13,8 mo
NCT03215810	Pretreated with ICIs	I	TILs	13	46%	92%	–	–
NCT03987867	First line	I	CIK cells + chemotherapy + Sintilimab (anti-PD-1)	32	81,3%	100%	6 mo-PFS 84,4%	–
Atalante (NCT02654587)	Pretreated with ICI and platinum chemo	III random	OSE2101 (anticancer vaccine) vs docetaxel/pemetrexed	118 pts PoI	8% vs 18%	6-mo DCR 26% vs 25%	2,7 vs 3,4 mo	11,1 vs 7,5 mo p=0,02

One phase I study in 20 NSCLC and ten melanoma patients pretreated with ICIs evaluated the efficacy of the combination of two antibodies directed to two different receptors of the TNF family, OX-40 and CD137, with an ORR of 5% and a DCR of 40% in the NSCLC cohort (63). Utomilumab, a CD137 agonist, was also tested in monotherapy during the dose-escalation phase I and with pembrolizumab, but the number of NSCLC patients was insufficient to formally evaluate efficacy (64, 65). An OX-40 agonist (GSK998) has been investigated in combination with pembrolizumab in heavily pretreated patients with different solid tumors, with relatively low efficacy (DCR 19%), but data regarding NSCLC patients are lacking (66)

One recent phase II study using an IL-15 superagonist (activating NK and CD-8 activity) in patients with different tumors having acquired resistance to ICIs, showed that this molecule (N803), given in combination with the same ICI in 135 patients (NSCLC 60%) has DCR of 59% and median PFS of 3,9 months with relatively low toxicity (67).

### Targeting Epigenetic Mechanisms

Histone deacetylase inhibitors may restore immunotherapy sensitivity in tumor cells by de-repressing the expression of MHC and co-stimulatory signals (34). The ENCORE 601 phase I/II trial showed an ORR of 19% in pretreated melanoma using entinostat in combination with an ICI (68). Another expansion cohort of ENCORE 601 with 71 NSCLC patients pretreated with ICIs showed an ORR of only 9,2% but encouraging activity in the responding subgroup of patients (median duration of response of 10.1 months) (69).

### Cellular and Vaccine Therapy

Adoptive cell therapy with tumor-infiltrating lymphocytes is effective in melanoma and is feasible in NSCLC, but its efficacy has not been established (20, 70). A phase I study with TILs in 20 NSCLC patients (30% PD-L1 >50%) resistant to nivolumab monotherapy recently showed a very good disease control rate of 92% and two ongoing complete responses in a total of 16 evaluable patients (71). Of note, toxicity seems manageable, with a severe toxicity rate of 12.5%.

A patient with NSCLC was treated with chimeric antigen receptor (CAR) T-cells directed against PD-L1, with significant pulmonary toxicity (72). Recently, nine patients with EGFR-positive NSCLC were treated with CAR T-cells directed against EGFR. Only one had a partial response to treatment, lasting more than 13 months (73).

Cytokine-induced killer (CIK) cells are derived from a mixture of T and NK cells isolated from the host’s peripheral blood and expanded *in vitro* with the use of specific stimulating cytokines and a CD3 antibody. This method is more rapid (14 days manufacturing) and less costly than the development of CAR T-cells, but standard quality control procedures are still lacking (74). Some interesting results were reported in patients with hematological malignancies and gastric cancer in China (75). A recent abstract of a phase I trial presented at ASCO 2021 showed promising results with the combination of CIK with first-line chemo-immunotherapy in 32 NSCLC patients, obtaining an impressive ORR of 81,3% (76).

Finally, the first randomized vaccination trial in patients with ICIs resistant NSCLC was presented at ESMO 2021. OSE2101 is an anticancer vaccine targeting five tumor neoantigens associated with HLA-A2. Unfortunately, the trial has been affected by the COVID-19 pandemic, which required multiple amendments. The presented preliminary results were derived from a smaller population of interest (118 patients) which comprised patients with secondary resistance to ICIs and pretreated sequentially with chemotherapy and ICIs. Somewhat surprisingly, despite the absence of significant PFS benefit, a statistically significant overall survival benefit (11,1 vs. 7,5 mo p=0,02) was observed (77).

### Targeting Tumor Microenvironment + Anti-PD-(L)1 Antibodies

Combining antiangiogenic TKIs with immunotherapy is an exciting strategy that has been demonstrated to be effective in endometrial, kidney cancer, and hepatocellular carcinoma (78–80). This approach has shown initially favorable results in NSCLC (**Table 3**).

**Table 3:** Results of clinical trial for new agents targeting Tumor Microenvironment (TME).

Trial name/code	Setting	Phase	Molecule	N° of NSCLC patients	ORR (CR)	DCR	Median PFS	Median OS
NCT02501096	Pretreated (52% with ICIs)	II	Pembrolizumab + Lenvatinib	21	33%	82%	5,9 mo	–
MRTX-500 (NCT02954991)	Pretreated with ICI (having had clinical benefit)	II	Nivolumab + sitravatinib (anti-VEGF et anti-TAM)	68 non squamous	16%	–	6 mo	15 mo
NCT03666143	Pretreated (63% with ICI)	II	Tislelizumab (anti-PD-1) + sitravatinib	75	17%	85%	5,5 mo	–
NCT03083041	First line	II	Apatinib + camrelizumab	25 non squamous	40%PD-L1+40%PD-L1-	92%	11 mo PD-L1 + 9,7 mo PD-L1-	NR
NCT03583086	Pretreated, ICI primary resistant	II	Vorolanib + nivolumab	15	7%	57%	–	–
NCT04646330	First line PD-L1+	I	AK104 (bispecific Ab vs PD-1 and CTLA-4) + anlotinib	8	62,5%	100%	–	–
NCT02517398	Pretreated, ICIs naive	I	Bintrafusp alfa (fusion protein anti PD-L+ & TGFβR)	80	21,3%	40%	–	–
NCT03774979	First line PD-L1+	I	SHR-1701 fusion protein anti PD-L1 & TGFβR	52	44,2%	73,1	–	–

The addition of lenvatinib, a multikinase inhibitor of VEGFR, FGFR, RET, and KIT, to pembrolizumab in 21 patients (11 pretreated with ICIs) showed encouraging results in a phase Ib/II multicohort study, with an ORR of 33% and a mPFS of 5,9 months. The safety profile was consistent with the association of the two drugs (81, 82). Vorolanib, a VEGF inhibitor that is structurally similar to sunitinib, was combined with nivolumab and showed encouraging efficacy, with a DCR of 57% in a primary refractory population of NSCLC patients treated in a phase II multicohort study (83).

Sitravatinib is a small molecule TKI targeting VEGF2 and TAM receptors on macrophages. It recently showed promising activity in combination with checkpoint inhibitors (84, 85). In particular, data from a phase II trial of sitravatinib and nivolumab in a population of 68 patients with non-squamous NSCLC with acquired resistance after an ICI-based regimen showed an ORR of 16% but a DCR of 78% and an impressive OS of 14,9 months with a 2-year survival rate of 32% (85). Based on these results, there is an ongoing phase III trial (SAPPHIRE, NCT03906071) with this association for NSCLC patients with acquired resistance to ICIs. Anlotinib, another multikinase inhibitor, showed responses but an excess of toxic deaths in squamous NSCLC patients (86). However, anlotinib combined with a bispecific anti-PD-1 and anti-CTLA-4 antibody did not provoke grade 4 or 5 toxicity among 18 first-line PD-L1 positive NSCLC patients, including nine with squamous histology. Moreover, ORR reached 62,5% and DCR 100% among eight evaluable patients (87). The combination of apatinib + camrelizumab (an anti-PD-1 antibody) showed a favorable efficacy profile in first-line non-squamous NSCLC in a small phase II study (88).

### Targeting Cytokines and Other Immunoregulatory Molecules

As discussed above, TGFβ plays an essential role in immune evasion through multiple actions on dendritic cells, Th1, Th2, and Tregs (9, 42). The development of therapies directed against TGFβ is difficult, often hindered by excessive cardiovascular toxicity, cutaneous reactions, and gastrointestinal inflammation, but several compounds have been tested, including three recent trials in NSCLC.

The first evaluated the efficacy of a novel antibody (NIS793) against TGFβ in combination with spartalizumab, an antibody against PD-1, in NSCLC patients resistant to previous anti-PD-1 therapy (89). While the toxicity profile seemed acceptable, with few treatment-related severe adverse events and no grade 5 adverse events in the cohort of 120 patients, the efficacy among the 20 patients with NSCLC has not yet been reported.

Bintrafusp alfa is a first-in-class fusion protein targeting TGFβ receptor and PD-L1, tested in 80 pretreated but ICI naïve NSCLC patients. An ORR of 21,3% and a DCR of 40% was reported, with the best response at the dosage of 1200 mg and in patients whose tumor had high PD-L1 expression (40). Early results from the phase III INTR@PID Lung 037 trial, assessing bintrafusp alfa versus pembrolizumab in front-line PD-L1 high NSCLC were disappointing, leading to the discontinuation of this phase III trial (NCT03631706) (90).

SHR-1701 is another fusion protein targeting the TGFβ receptor and PD-L1, tested in a phase I trial in the first-line metastatic setting in PD-L1 positive NSCLC. In 52 patients, the authors reported an ORR of 44,2% and a DCR of 73,1% overall, with the best responses obtained in patients with tumors expressing PD-L1 ≥50% (91).

Other immunoregulatory cytokines beyond TGFβ are also being studied. IDO1 (indoleamine 2,3-dioxygenase) is an enzyme involved in tryptophan metabolism which influences T-cells’ activity. The efficacy of epacadostat, an IDO1 inhibitor, was not confirmed in a phase III study, despite promising results in early clinical studies (20).

### Chemotherapy and Radiotherapy

Last but not least, combining chemotherapy with immunotherapy is already a standard of care in lung cancer (NSCLC and SCLC), even if the mechanisms involved are not so clear (6). A potential synergy could be attributed to the release of tumor neoantigen *via* chemo-induced cell death and the upregulation of MHC-I (9).

The ability of radiotherapy to induce systemic immune changes and an effect at a distance, often called abscopal, is debated. Irradiation of lesions in oligoprogressive disease is a well-established standard of care for oncogene-addicted NSCLC but less established for patients on immunotherapy (92, 93). One prospective study for 50 patients with NSCLC and melanoma with progression of at most five lesions during ICIs treatment evaluated the efficacy of stereotactic radiotherapy, obtaining an ORR of 42%, mPFS of 14.2 months, and an impressive median OS of 37.4 months. A measurable abscopal effect was found in 40/50 patients with target lesions outside radiotherapy fields. Of these, 26 (65%) presented an abscopal response at two months (94).

## Future Directions

The treatment of NSCLC is rapidly evolving, as novel checkpoint inhibitors and treatment combinations are being identified.

The development of new compounds will enlarge the repertoire of immune checkpoints that can be targeted and dramatically increase the potential for treatment combinations with chemotherapy or antiangiogenic treatment or with other checkpoint inhibitors. The results of several large trials with new therapeutics are expected in the following years. Data from SKYSCRAPER-01 trial (NCT04294810) are awaited to assess tiragolumab, an anti-TIGIT antibody, with atezolizumab in first-line PD-L1 high NSCLC patients. The use of relatlimab (anti-LAG3) seems promising based on results obtained in melanoma and an interesting randomized phase II study is ongoing in NSCLC (NCT04623775).

In addition to new checkpoints, new therapeutics acting on well-known targets such as PD1/PDL1 or CTLA4, may prove more effective or less toxic. For example, ipilimumab-NF (BMS-986218) is a next-generation anti-CTLA4 antibody with increased activity in preclinical studies that is currently being tested alone or in combination with nivolumab in various tumors, including NSCLC (NCT03110107). Furthermore, combinations of immunotherapy with TKIs, already shown to be efficacious in other cancer types, are being tested. For example, the SAPPHIRE trial (NCT03906071) which randomizes patients progressing after chemo-immunotherapy to sitravatinib, an inhibitor of multiple tyrosine kinases (RET, TAM family receptors, VEGFR2, KIT), with nivolumab versus docetaxel, could establish a new standard for patients with acquired resistance to anti-PD-1.

Finally, cellular therapy with TILs, CAR T-cells, or CIK may prove to be effective, but their development is limited to very few highly specialized centers and remains costly and highly complex. Bispecific antibodies seem to be an attractive solution that can attract T-cells to cancer cells expressing specific protein targets. A PSMAxCD3 bispecific antibody is being tested in squamous lung cancer (NCT04496674)

It is not yet clear which of these strategies will prove to be superior in the long run. The arrival of new immune-based therapies should ultimately increase patient benefit, including both the proportion of patients who derive response and the duration of response. New drugs may have greater efficacy, new combinations of existing drugs may overcome resistance to immunotherapy or it may even become possible to choose the most appropriate immunotherapy for each individual tumor, based on tumor and patient characteristics.

At the same time, basic research is moving towards a more and more specific analysis of tumors and TME, especially with the use of techniques such as single-cell RNA sequencing (95). This has led to the first characterization of the complex and heterogeneous landscape of NSCLC in a recent analysis of 42 biopsy samples (96). Further research on this direction could clarify the interactions between tumor and TME, also highlighting the role of important cell subpopulations such as tumor-infiltrating neutrophils that may contribute to resistance and represent promising targets (97).

## Conclusions

ICIs have become an essential component of front-line systemic treatment in combination with chemotherapy or in monotherapy. Primary ICI resistance remains common, and secondary resistance appears within two years for most patients, highlighting the need for more effective treatment. Results from many preclinical studies using novel ICI combinations and/or targeted therapies have fueled multiple clinical trials in the last few years. Hopefully, this vigorous research activity will translate to even more favorable patient outcomes in the near future.

## Author Contributions

All authors listed have made a substantial, direct, and intellectual contribution to the work, and approved it for publication.

## Funding

PT research activity is funded by Ligue Genevoise Contre le Cancer (Fonds de soutien de la recherche translationnelle en hémato-oncologie). Open access funding was provided by the University of Geneva.

## Conflict of Interest

AF: Advisory board with Roche, Pfizer, Astellas, AstraZeneca, MSD, Sanofi, Novartis and BMS. AA: Advisory board: MSD Oncology, Roche, Takeada, Pfizer, Bristol-Myers Squibb, AstraZeneca, Eli-Lilly, Roche. Speaker Bureau: Eli-Lilly, AstraZeneca.PT: Advisory board with Astellas, Bayer, BMS, Ipsen, Janssen-Cilag, Merck, MSD, Pfizer, Roche and Sanofi. Travel and conference expenses from Lilly, Janssen-Cilag and Sanofi.

The remaining author declares that the research was conducted in the absence of any commercial or financial relationships that could be construed as a potential conflict of interest.

## Publisher’s Note

All claims expressed in this article are solely those of the authors and do not necessarily represent those of their affiliated organizations, or those of the publisher, the editors and the reviewers. Any product that may be evaluated in this article, or claim that may be made by its manufacturer, is not guaranteed or endorsed by the publisher.
