# Erratum: The landscape of immunotherapy resistance in NSCLC

**DOI:** 10.3389/fonc.2023.1187021

**Published:** 2023-04-04

**Authors:** Frontiers Production Office

**Affiliations:** Frontiers Media SA, Lausanne, Switzerland

**Keywords:** immunotherapy, resistance, checkpoint inhibitor, NSCLC, lung cancer

An omission to the funding section of the original article was made in error. The following sentence has been added: “Open access funding was provided by the University of Geneva”.

The original version of this article has been updated.

